# Misidentification of *Plasmodium ovale* as *Plasmodium vivax* malaria by a microscopic method: a meta-analysis of confirmed *P. ovale* cases

**DOI:** 10.1038/s41598-020-78691-7

**Published:** 2020-12-11

**Authors:** Manas Kotepui, Frederick Ramirez Masangkay, Kwuntida Uthaisar Kotepui, Giovanni De Jesus Milanez

**Affiliations:** 1 Medical Technology, School of Allied Health Sciences, Walailak University, Tha Sala, Nakhon Si Thammarat, Thailand; 2 Department of Medical Technology, Institute of Arts and Sciences, Far Eastern University-Manila, Manila, Philippines

**Keywords:** Malaria, Epidemiology

## Abstract

*Plasmodium ovale* is a benign tertian malaria parasite that morphologically resembles *Plasmodium vivax*. *P. ovale* also shares similar tertian periodicity and can cause relapse in patients without a radical cure, making it easily misidentified as *P. vivax* in routine diagnosis. Therefore, its prevalence might be underreported worldwide. The present study aimed to quantify the prevalence of *P. ovale* misidentified as *P. vivax* malaria using data from studies reporting confirmed *P. ovale* cases by molecular methods. Studies reporting the misidentification of *P. ovale* as *P. vivax* malaria were identified from three databases, MEDLINE, Web of Science, and Scopus, without language restrictions, but the publication date was restricted to 1993 and 2020. The quality of the included studies was assessed using the Quality Assessment of Diagnostic Accuracy Studies (QUADAS). The random-effects model was used to estimate the pooled prevalence of the misidentification of *P. ovale* as *P. vivax* malaria by the microscopic method when compared to those with the reference polymerase chain reaction method. Subgroup analysis of participants was also performed to demonstrate the difference between imported and indigenous *P. ovale* cases. The heterogeneity of the included studies was assessed using Cochran's Q and I^2^ statistics. Publication bias across the included studies was assessed using the funnel plot and Egger’s test, and if required, contour-enhanced funnel plots were used to identify the source(s) of funnel plot asymmetry. Of 641 articles retrieved from databases, 22 articles met the eligibility criteria and were included in the present study. Of the 8,297 malaria-positive cases identified by the PCR method, 453 *P. ovale* cases were confirmed. The pooled prevalence of misidentification of *P. ovale* as *P. vivax* malaria by the microscopic method was 11% (95% CI: 7–14%, I^2^: 25.46%). Subgroup analysis of the participants demonstrated a higher prevalence of misidentification in indigenous cases (13%, 95% CI: 6–21%, I^2^: 27.8%) than in imported cases (10%, 95% CI: 6–14%, I^2^: 24.1%). The pooled prevalence of misidentification of *P. vivax* as *P. ovale* malaria by the microscopic method was 1%, without heterogeneity (95% CI: 0–3%, I^2^: 16.8%). PCR was more sensitive in identifying *P. ovale* cases than the microscopic method (p < 0.00001, OR: 2.76, 95% CI: 1.83–4.15, I^2^: 65%). Subgroup analysis of participants demonstrated the better performance of PCR in detecting *P. ovale* malaria in indigenous cases (p: 0.0009, OR: 6.92, 95% CI: 2.21–21.7%, I^2^: 68%) than in imported cases (p: 0.0004, OR: 2.15, 95% CI: 1.41–3.29%, I^2^: 63%). *P. ovale* infections misidentified as *P. vivax* malaria by the microscopic method were frequent and led to underreported *P. ovale* cases. The molecular identification of *P. ovale* malaria in endemic areas is needed because a higher rate of *P. ovale* misidentification was found in endemic or indigenous cases than in imported cases. In addition, updated courses, enhanced training, and refreshers for microscopic examinations, particularly for *P. ovale* identification, are necessary to improve the microscopic identification of *Plasmodium* species in rural health centres where PCR is unavailable.

## Introduction

*Plasmodium ovale* is one of the five *Plasmodium* species that can infect humans, namely: *P. falciparum*, *P. vivax*, *P. malariae*, *P. knowlesi*, and *P. ovale*^1^. It is endemic to tropical western Africa and the Southwest Pacific but rarely occurs outside of these regions, with less than 1% isolates^2^. The rarity of *P. ovale* infections in published studies might be due to the low species-specific parasitaemia and the short duration of patient infections^3^. Compared to the other four *Plasmodium* species that can infect humans, *P. ovale* has morphological characteristics and the ability to cause relapse, similar to *P. vivax*. *P. ovale* malaria does not usually cause severe malaria, but a study of 1365 *P. ovale* malaria cases demonstrated that 3% of severe malaria cases were caused by *P. ovale* infection, including jaundice (1.1%), severe anaemia (0.88%), and pulmonary impairments (0.59%)^4^. The ability to cause relapse by way of hypnozoites in the liver is recognized in both *P. ovale* and *P. vivax* malaria and leads to the reactivation of dormant liver stages weeks or months later^1^.

The routine diagnostic method of patients suspected of having *P. ovale* infection is the visualization of thick and thin blood smears by microscopy and/or rapid diagnostic tests (RDT), if available^5^. It is well documented that microscopy is an inexpensive and rapid quantification of parasites and is a relatively sensitive method; nevertheless, it has several limitations; it is time consuming, it misdiagnoses *Plasmodium* species, it requires expert or well-trained microscopists, and it misses *Plasmodium* species in case of a low parasite density in mixed infection^6,7^. Since *P. ovale* infects only young erythrocytes, the parasite density is low, and infection with other *Plasmodium* species is mixed^8^, resulting in missed identification by microscopists. The advantages of the recent molecular technique in identifying *P. ovale*, polymerase chain reaction (PCR), which amplifies 18S small subunit ribosomal RNA (18S rRNA) target genes offers high sensitivity and high specificity^9^. Moreover, two distinct *P. ovale* subspecies, *P. ovale curtisi* (classic type) and *P. ovale wallikeri* (variant type), occur globally^10^. No differences in the clinical or laboratory characteristics or other demographic data were summarized. Some studies demonstrated that higher parasite density, platelet counts, and latency periods were reported in *P. ovale curtisi* infection than in *P. ovale wallikeri* infection^11,12^.

Routine identification of *P. ovale* relies on blood smear examination, which can lead to underestimation of the true prevalence of *P. ovale* globally, with little clinical consequence of the misidentification of *P. ovale* as *P. vivax*. Molecular techniques such as PCR were used to identify *P. ovale* to prevent confusion with *P. vivax* or to prevent missed identification of *P. ovale* mixed infection with other *Plasmodium* species, such as mixed infection with *P. falciparum* or with *P. knowlesi*. Mixed infections of *Plasmodium* spp. could lead to severe malaria if the treatment was inadequate or incorrect^13^; therefore, it is also necessary to detect sub-microscopic mixed infection of *P. ovale* by molecular methods. The present study aimed to quantify the microscopic misidentification of *P. ovale* as *P. vivax* to support and promote the use of molecular techniques for the accurate identification of *P. ovale* malaria and promote a training course on *P. ovale* microscopic identification by health governors to increase the accuracy of *P. ovale* identification by microscopic methods.

## Methods

### Report guideline of the systematic review

The protocol of this study was registered at the International Prospective Register of Systematic Reviews (PROSPERO)^14^ with registration number CRD42020204049. The report of this systematic review and meta-analysis followed the Preferred Reporting Items for Systematic Reviews and Meta-Analyses (PRISMA) guidelines (Checklist S1)^15^**.**

### The search strategy

Searches were performed in three databases that included MEDLINE, Scopus, and Web of Science, without language restrictions, but the publication date was restricted to 1993 and 2020. The search terms used are provided in Table S1. Searches of the reference list of the final included studies and review articles were also performed to reduce the chance of missing relevant studies.

### Eligibility criteria

Observational studies that reported the prevalence of both *P. vivax* and *P. ovale* malaria by microscopy and PCR or molecular methods were screened for the misidentification of *P. ovale* as *P. vivax* malaria by a microscopic method. The inclusion criteria were (1) studies reporting the prevalence of the misidentification of *P. ovale* as *P. vivax* malaria by a microscopic method and (2) studies using PCR or molecular methods to confirm the *Plasmodium* species. The exclusion criteria were (1) case reports or case series that reported a small number of patients, which can lead to reporting bias for meta-analysis, (2) studies using both microscopic and molecular methods to identify *Plasmodium* species where the data could not be extracted, (3) studies carried out on the performance of tests as those tests attempted to develop new techniques or new tests for the detection of *Plasmodium* species, (4) experimental studies that aimed to explore the new finding related to *Plasmodium* species, (5) studies with no misidentification of *P. ovale* to *P. vivax,* or no *P. vivax* malaria was observed as those studies did not provide the evidence of the misidentification of *P. ovale* as *P. vivax* malaria, (6) review articles, (7) studies without the full text, (8) clinical trials, guidelines, studies using the same participants, and other studies without relevant data.

### Study selection and data extraction

Two authors (MK and FRM) selected potentially relevant studies according to the eligibility criteria. Any discordance in the study selection was resolved by consensus. Data selection from relevant studies was managed using Endnote software X7 (Clarivate Analytics, Philadelphia, USA). Data extraction was also performed by two authors (MK and FRM) and crosschecked by the third author (KUK). The following data were extracted: name of first author, year of publication, study area, years of the study, study design, age range (years), gender (male, %), participants (imported or indigenous), PCR for identified *Plasmodium* spp., target gene for PCR, number of malaria cases identified by microscopy and PCR methods, number of *P. vivax* cases identified by microscopy and PCR methods, number of *P. ovale* cases identified by microscopy and PCR methods, number of misidentifications of *P. ovale* as *P. vivax* malaria by microscopy, and number of misidentifications of *P. vivax* as *P. ovale* malaria by microscopy. The data were extracted to pilot-standardized sheets created using Microsoft Excel 2010 (Microsoft Corporation, Washington, USA) before meta-analyses.

### Quality of the included studies

The quality of the individual studies was assessed using the Quality Assessment of Diagnostic Accuracy Studies (QUADAS) (Table S2)^16^. The tool was comprised of 4 domains: patient selection, index test, reference standard, and flow and timing. Each domain was assessed in terms of risk of bias and concerns of applicability^16^. The index test was microscopy, while the reference standard was PCR method. The results of the QUADAS of all included studies were summarized in the methodological quality graph and summary.

### Outcomes

The primary outcome of the present study was the prevalence of misidentification of *P. ovale* as *P. vivax*, and also *P. vivax* as *P. ovale* malaria by the microscopic method. The secondary outcome was the performance of the PCR test to identify *P. ovale* malaria compared to that of the microscopic method.

### Data synthesis

For the primary outcome, the pooled prevalence of misidentification of *P. ovale* as *P. vivax*, and also* P. vivax* as *P. ovale* malaria by the microscopic method was estimated using a random-effects model provided by STATA Statistical Software version 15.0 (StataCorp. 2017. Stata Statistical Software: Release 15. College Station, TX: StataCorp LLC). The number of *P. ovale* misidentified as *P. vivax* by microscopy and the total number of true *P. ovale* malaria identified by PCR were computed using the “metaprop case total case” command provided in STATA Statistical Software version 15.0. For the secondary outcome, the performance of PCR to identify *P. ovale* malaria compared to that of the microscopic method, was estimated using the random-effects model provided by Review Manager 5.3 (The Cochrane Collaboration, London, UK) available at https://training.cochrane.org/. The results of primary and secondary outcomes were demonstrated as pooled prevalence or odds ratios (ORs) with their 95% confidence intervals (CIs) in the forest plot. Heterogeneity across the included studies was assessed using Cochran's Q and I^2^ statistics. Subgroup analyses of participants (imported or indigenous cases) were performed to identify the difference in the population that might affect the misidentification of *P. ovale* as *P. vivax* malaria.

### Publication bias

Publication bias among the included studies was assessed by visualization of the funnel plot asymmetry. Funnel plot asymmetry was also assessed by Egger's test to quantify publication bias if it existed.

### Consent for publication

All authors have read the manuscript and consent to its publication.

## Results

### Search results

**Figure 1:**
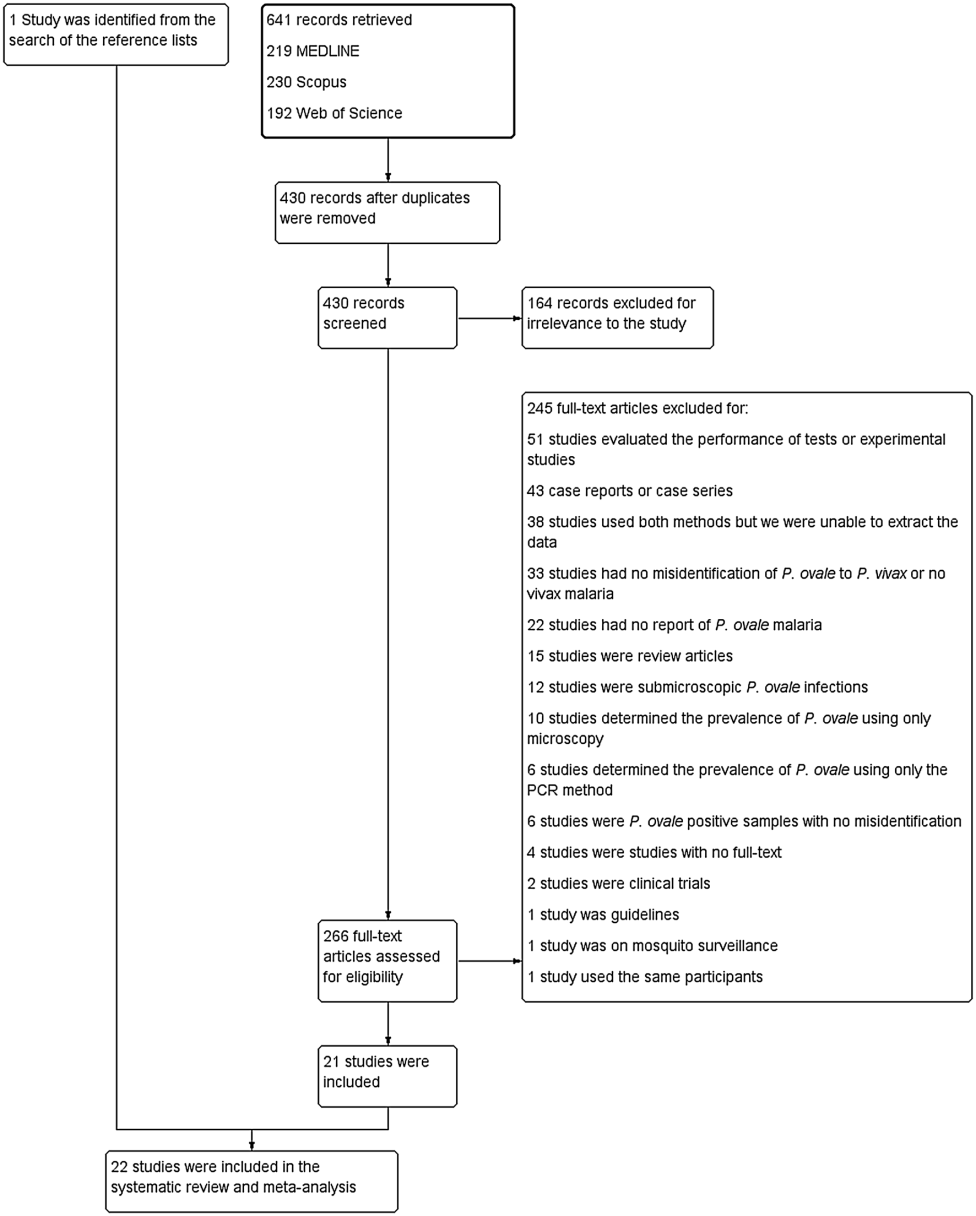
Flow chart for study selection.

The literature search identified 641 records through three different databases: 219 for MEDLINE, 230 for Scopus, and 192 for Web of Science (Fig. 1). After the removal of duplicate articles, the remaining 430 studies were screened. In total, 164 records were excluded because they were irrelevant. The 266 potentially relevant studies were assessed in detail and 245 studies were excluded for the following reasons: 51 studies evaluated the performance of tests or experimental studies, 43 were case reports or case series, 38 studies used both methods but we were unable to extract the data, 33 had no misidentification of *P. ovale* to *P. vivax* or no vivax malaria, 22 had no report of *P. ovale* malaria, 15 were review articles, 12 were sub-microscopic *P. ovale* infections, 10 determined the prevalence of *P. ovale* using only microscopy, 6 determined the prevalence of *P. ovale* using only the PCR method, 6 were *P. ovale* positive samples with no misidentification, 4 were studies had no full-text, 2 were clinical trials, 1 was guidelines, 1 was on mosquito surveillance, and 1 study used the same participants. Twenty-one studies^17–37^ were included because they met the eligibility criteria, and one additional study^38^ was selected after reviewing the reference lists of the 20 included studies and review articles. Finally, a total of 22 studies^17–38^ were included for qualitative and quantitative syntheses.

### Characteristics of the included studies

All characteristics of the included studies can be found in Table 1. Twenty-two studies reported evidence of *P. ovale* misidentification as *P. vivax* by a microscopic method between 1993 and 2020. Of the 22 included studies, 8 studies^20,23,26,27,29,33,35,37^ reported evidence of *P. vivax* misidentification as *P. ovale* by the microscopic method. Most of the included studies were observational cross-sectional studies (17/22, 77.3%). Most of the studies (8/22, 36.4%) were conducted in Asia (2 in China^34,36^, 2 in Thailand^25,30^, Singapore^38^, Israel^23^, Sri Lanka^24^, and Malaysia^35^), Europe (7/22, 31.8%) (3 Italy^18,28,29^, 2 Belgium^27,37^, Germany^22^, and Switzerland^32^), America (4/22, 18.2%) (3 United States^19,20,31^, Canada^26^), Africa (2/22, 9.1%) (2 Ethiopia^17,21^), and Oceania (1/22, 4.55%) (1 Australia^33^). Most of the included studies (13/22, 59.1%) identified *P. ovale* using blood samples from patients suspected of having malaria. Almost half of the included studies (10/22, 45.5%) reported using nested PCR targeting 18S rRNA for identifying *Plasmodium* parasites, while the remaining studies used real-time PCR or did not specify the type of PCR. Twenty-two studies reported that a total of 8079 malaria cases were identified by microscopic methods, while a total of 8297 malaria cases were identified by PCR. A total of 453 *P. ovale* cases were confirmed by the PCR method, while 204 *P. ovale* cases were first identified by the microscopic method and subsequently confirmed as *P. ovale* by the PCR method. The misidentification of *P. vivax* and other *Plasmodium* species is shown in Table 2.

**Table 1:** Characteristics of the included studies.

No	Author, year	Study area (years of the survey)	Study design	Age range (years)	Gender (male, %)	Participants	Molecular techniques for *Plasmodium* sp.	Target gene	Microscopy (include mixed infection)			PCR/Molecular techniques (include mixed infection)			No. of *P. ovale* as *P. vivax*	No. of *P. vivax* as *P. ovale*
									No. malaria	No. of *P. vivax*	No. of *P. ovale* (n/N)*	No. malaria	No. of *P. vivax*	No. of *P. ovale*		
1	Alemu et al., 2014	Ethiopia (2013)	Cross sectional study	NS	NS	297 patients with suspected malaria	Nested PCR	18S rRNA	183	51	0	217	68	9	4	0
2	Calderaro et al., 2013	Italy (2000–2012)	Retrospective cross-sectional study	NS	PCR positive, 82 (64%)	398 patients with suspected malaria	Real-time PCR	18S rRNA	126	9	8 (7/8)	128	7	14	1	0
3	Chavatte et al., 2015	Singapore (2001–2014)	Retrospective cross-sectional study	NS	NS	1053 malaria positive	Real-time PCR	18S rRNA	1053	NS	0	1053	NS	11	11	0
4	Cullen et al., (2014)	USA (2012)	Retrospective cross-sectional study	NS	NS	104 malaria positive for genetic markers	NS	18S rRNA	104	9	7 (5/7)	104	14	12	1	0
5	Cullen et al., (2016)	USA (2013)	Retrospective cross-sectional study	NS	NS	137 malaria positive for genetic markers	NS	18S rRNA	137	8	5 (2/5)	137	15	14	1	1
6	Díaz et al., 2015	Ethiopia (2010–2011)	Cross sectional study	Mean 13.4 (1–80), median 10	1507	3060 patients with suspected malaria, for microscopy and 1209 for PCR	Semi-nested multiplex PCR	Cytochrome b	736	436	0	788	398	24	2	0
7	Frickmann et al., 2019	Germany (2010–2019)	Retrospective cross-sectional study	31.6 ± 14.8	56, 72.7%	77 *P. ovale* positive cases	Real-time PCR	18S rRNA and Po-ldh	77	16	25 (25/25)	77	0	77	3	0
8	Grossman et al., 2016	Israel (2009–2015)	Cross-sectional study	NS	NS	357 patients with suspected malaria	Real-time PCR	18S rRNA	307	73	7 (2/7)	288	104	23	3	4
9	Gunasekera et al., 2018	Sri Lanka (2014–2017)	Cross-sectional study	PCR positive 37 (1–66)	PCR positive 159, 91.9%	350 patients with suspected malaria	Nested PCR	18S rRNA	164	77	9 (9/9)	173	77	10	1	0
10	Han et al., 2007	Thailand	Retrospective cross-sectional study	NS	NS	121 malaria positive and negative cases	Nested PCR	18S rRNA	68	34	5 (5/5)	71	10	8	2	0
11	Humar et al., 1997	Canada (1993–1995)	Cross-sectional study	NS	NS	182 patients with suspected malaria	Nested PCR	18S rRNA	159	87	11 (10/11)	159	88	15	3	1
12	Loomans et al., 2019	Belgium (2013–2017)	Cross-sectional study	Median (36, 1–84)	610, 64.4%	947 malaria positive and negative cases	Real-time PCR	18S rRNA	927	77	46 (27/46)	893	81	63	8	3
13	Maltha et al., 2010	Belgium (1996–2009)	Retrospective cross-sectional study	35 (1–84)	2.16:1	590 malaria positive and negative cases	NS	18S rRNA	495	79	73 (69/73)	495	76	76	7	4
14	Paglia et al., 2012	Italy (1998–2003)	Cross-sectional study	Malaria positive 38 ± 12	2:1	1226 patients with suspected malaria	Semi-nested PCR	18S rRNA	187	17	4 (3/4)	196	20	7	2	0
15	Perandin et al., 2004	Italy	Retrospective study	NS	NS	122 patients with suspected malaria	Nested PCR	18S rRNA	61	12	3 (2/3)	60	8	10	5	1
16	Putaporntip et al., 2009	Thailand (2006–2007)	Cross-sectional study	Median (23, 1–81)	2.25:1	1874 patients with suspected malaria	Nested PCR	18S rRNA	1695	1013	0	1751	1192	18	1	0
17	Reller et al., 2013	USA (2004–2012)	Cohort study	NS	NS	148 malaria positive	Multiplex quantitative real-time PCR	18S rRNA	146	38	17 (17/17)	157	37	20	2	0
18	Rougemont et al., 2004	Switzerland (2002–2003)	Prospective study	NS	NS	60 patients with suspected malaria	Real-time PCR	18S rRNA	31	4	3 (2/3)	34	5	4	1	0
19	Whiley et al., 2004	Australia	Prospective study	NS	NS	279 patients with suspected malaria	Nested PCR	18S rRNA	219	131	6 (5/6)	225	131	6	1	1
20	Xu et al., 2016	China (2012–2014)	Cross-sectional study	20–54 (96.8%)	92.5:1	374 patients with suspected malaria	Nested PCR	18S rRNA	374	40	14 (14/14)	364	44	16	2	0
21	Yusof et al., 2014	Malaysia (2012–2013)	Retrospective study	NS	77.9%	457 malaria positive	Nested PCR	18S rRNA	457	137	1 (0/1)	543	144	2	2	1
22	Zhou et al., 2014	China (2008–2012)	Cross-sectional study	NS	NS	562 patients with suspected malaria	Nested PCR	18S rRNA	373	275	0	384	288	14	4	0

*NS* not specified, *n/N number of *P. ovale* cases confirmed by PCR/number of *P. ovale* cases detected by microscopy.

**Table 2:** Misidentification of any *Plasmodium* species such as *P. ovale* by microscopic method.

No	Author, year	Microscopy	PCR/molecular techniques		
		No. of *P. ovale*	True *P. ovale* cases	Number of misidentifications	Misidentified *Plasmodium* species
1	Alemu et al., 2014	0	–	–	–
2	Calderaro et al., 2013	8 (7/8)	7	1	*P. falciparum*
3	Chavatte et al., 2015	0	–	–	–
4	Cullen et al., 2014	7 (5/7)	5	2	1 *P. falciparum,* 1 *P. malariae*
5	Cullen et al., 2016	5 (2/5)	2	3	2 *P. falciparum*, 1 *P. vivax*
6	Díaz et al., 2015	0	–	–	–
7	Frickmann et al., 2019	25 (25/25)	25	0	–
8	Grossman et al., 2016	7 (2/7)	2	5	4 *P. vivax*, 1 *P. falciparum* + *P. vivax*
9	Gunasekera et al., 2018	9 (9/9)	9	0	–
10	Han et al., 2007	5 (5/5)	5	0	–
11	Humar et al., 1997	11 (10/11)	10	1	1 *P. vivax*
12	Loomans et al., 2019	46 (27/46)	27	19	10 *P. falciparum,* 3 *P. vivax,* 6 *P. malariae*
13	Maltha et al., 2010	73 (69/73)	69	4	4 *P. vivax*
14	Paglia et al., 2012	4 (3/4)	3	1	1 *P. falciparum*
15	Perandin et al., 2004	3 (2/3)	2	1	1 *P. vivax*
16	Putaporntip et al., 2009	0	–	–	–
17	Reller et al., 2013	17 (17/17)	17	0	–
18	Rougemont et al., 2004	3 (2/3)	2	1	1 *P. falciparum*
19	Whiley et al., 2004	6 (5/6)	5	1	1 *P. vivax*
20	Xu et al., 2016	14 (14/14)	14	0	–
21	Yusof et al., 2014	1 (0/1)	0	1	1 *P. vivax*
22	Zhou et al., 2014	0	–	–	–

### Quality of the included studies

**Figure 2:**
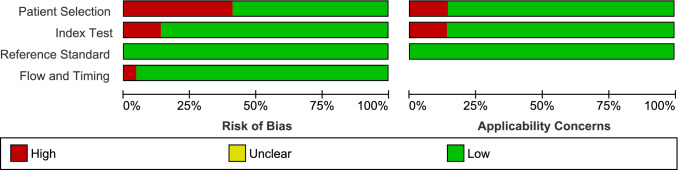
Methodological quality graph.

The quality of the individual studies assessed using QUADAS can be referenced in Fig. 2 and Supplementary Fig. 1.

### The pooled prevalence of the misidentification of *P. ovale* as *P. vivax* malaria

**Figure 3:**
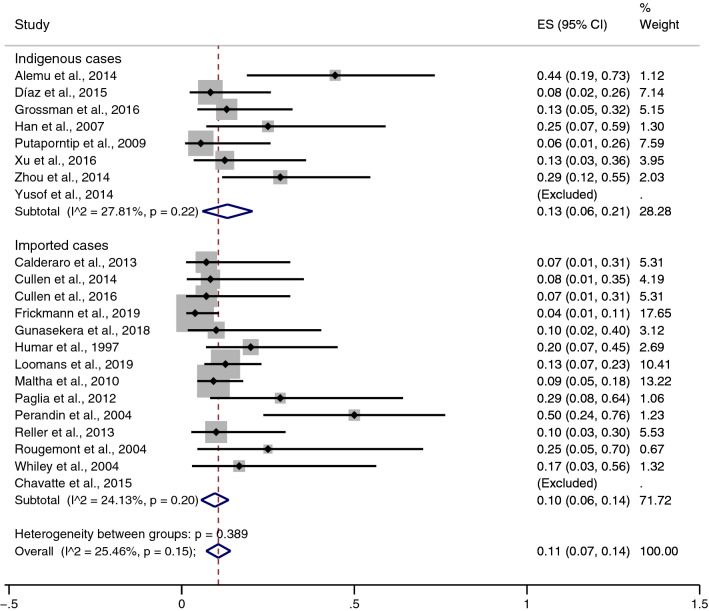
Pooled prevalence of misidentification of *P. ovale* as *P. vivax* malaria. ES: Estimated prevalence. The pooled prevalence was estimated using STATA Statistical Software version 15.0 (StataCorp. 2017. Stata Statistical Software: Release 15. College Station, TX: StataCorp LLC).

The pooled prevalence of the misidentification of *P. ovale* as *P. vivax* malaria was estimated using all 22 included studies (Fig. 3). Overall, the pooled prevalence of misidentification of *P. ovale* as *P. vivax* malaria by the microscopic method was 11% without heterogeneity (95% CI: 7–14%, I^2^: 25.5%). The prevalence of misidentification of *P. ovale* as *P. vivax* malaria in two studies^35,38^ was not estimated because both studies reported 100% misidentification. Subgroup analysis of participants demonstrated a higher prevalence of misidentification in indigenous cases (13%, 95% CI: 6–21%, I^2^: 27.8%) than in imported cases (10%, 95% CI: 6–14%, I^2^: 24.1%). The highest rate of misidentification in indigenous cases (44%, 95% CI: 79–43%) was demonstrated in the study by Alemu et al.^17^, while the highest rate of misidentification in imported cases (50%, 95% CI: 24–76%) was demonstrated in the study by Perandin et al.^29^.

### The pooled prevalence of the misidentification of* P. vivax *as *P. ovale* malaria

**Figure 4:**
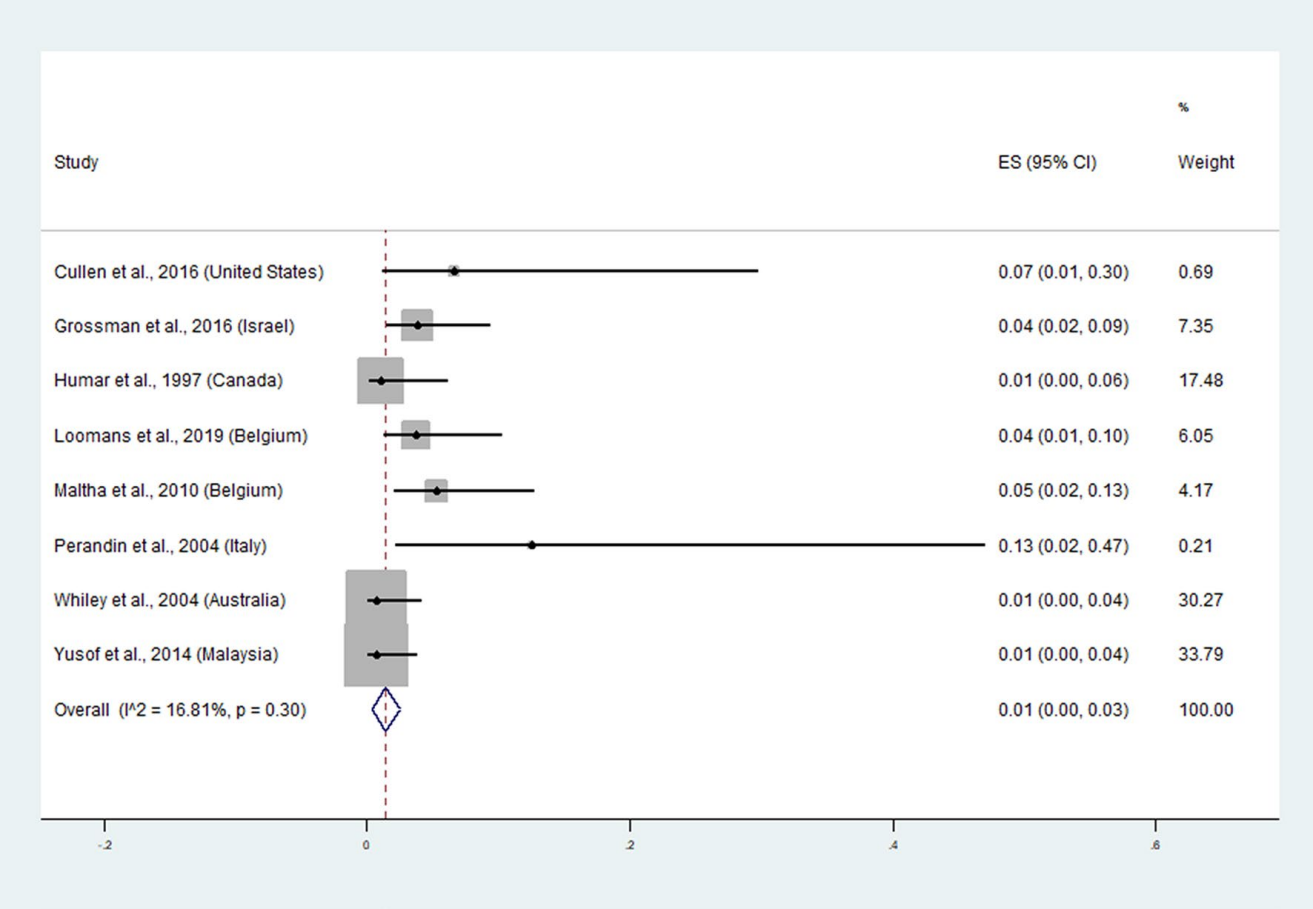
Pooled prevalence of misidentification of *P. vivax* as *P. ovale* malaria. ES: Estimated prevalence. The pooled prevalence was estimated using STATA Statistical Software version 15.0 (StataCorp. 2017. Stata Statistical Software: Release 15. College Station, TX: StataCorp LLC).

The pooled prevalence of the misidentification of *P. vivax* as *P. ovale* malaria was estimated using the data from 8 studies^20,23,26,27,29,33,35,37^. Overall, the pooled prevalence of misidentification of *P. vivax* as *P. ovale* malaria by the microscopic method was 1% without heterogeneity (95% CI: 0–3%, I^2^: 16.8%) (Fig. 4). A high rate of misidentification was reported in imported cases in Italy (13%, 95% CI: 2–47%)^29^, the United States (7%, 1–30%)^20^, Belgium (5%, 2–13%)^27^, Belgium (4%, 1–10%)^37^, and Israel (4%, 2–9%)^23^.

### The performance of PCR to identify *P. ovale* malaria compared to that of the microscopic method

**Figure 5:**
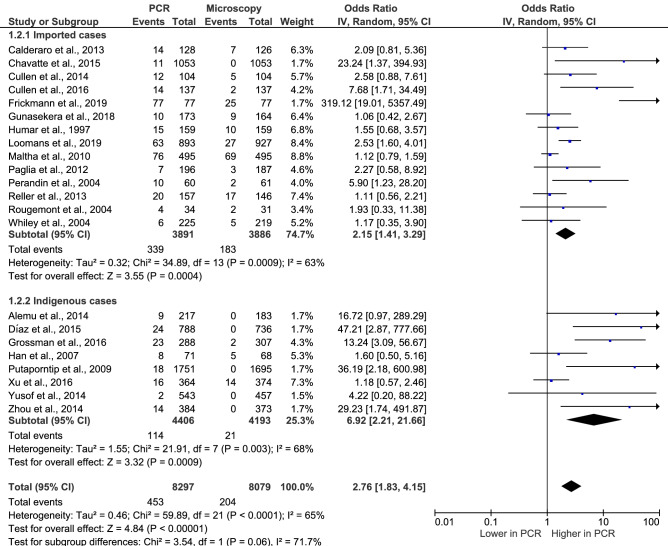
The performance of PCR to identify *P. ovale* IV: Inverse Variance, CI: Confidence Interval, Event: number of patients with *P. ovale*, random: random effects model, Total: number of all *P. ovale* cases, Lower in PCR: the proportion of *P. ovale* cases detected by PCR was lower than those detected by the microscopic method. Higher in PCR: the proportion of P. ovale cases detected by PCR was higher than those detected by the microscopic method. The performance of PCR to identify *P. ovale* malaria was analysed using Review Manager 5.3 (The Cochrane Collaboration, London, UK) available at https://training.cochrane.org/.

The performance of PCR to identify *P. ovale* malaria versus that of the microscopic method was estimated using the random-effects model (Fig. 5). The number of *P. ovale* cases identified using microscopic method that were subsequently confirmed by PCR method (204 cases) and the number of *P. ovale* identified by PCR were used in the present analysis. The results demonstrated a higher performance of PCR in identifying *P. ovale* cases than that of the microscopic method, with substantial heterogeneity (p < 0.00001, OR: 2.76, 95% CI: 1.83–4.15, I^2^: 65%). Subgroup analysis of participants demonstrated a higher performance of PCR in detecting *P. ovale* malaria in indigenous cases (p: 0.0009, OR: 6.92, 95% CI: 2.21–21.6, I^2^: 68%) than in imported cases with substantial heterogeneity (p: 0.0004, OR: 2.15, 95% CI: 1.41–3.29, I^2^: 63%). No difference between the two subgroups was found (p: 0.06, I^2^: 71.7%). Among indigenous cases, six included studies^17,21,30,35,36,38^ demonstrated no cases of *P. ovale* by the microscopic method and were the same cases that were confirmed by the PCR method.

### Sensitivity test

The robustness of the pooled prevalence of the misidentification of *P. ovale* as *P. vivax* malaria was determined using the trim and fill method by excluding low-quality studies from the pooled analysis. The result of the trim and fill method by removing the three studies^19,20,37^ with low qualities demonstrated that the pooled prevalence of the misidentification of *P. ovale* as *P. vivax* was similar to that of the pooled prevalence of 22 studies (11%, 95% CI: 7–16%, I^2^: 34.6%).

### Publication bias

**Figure 6:**
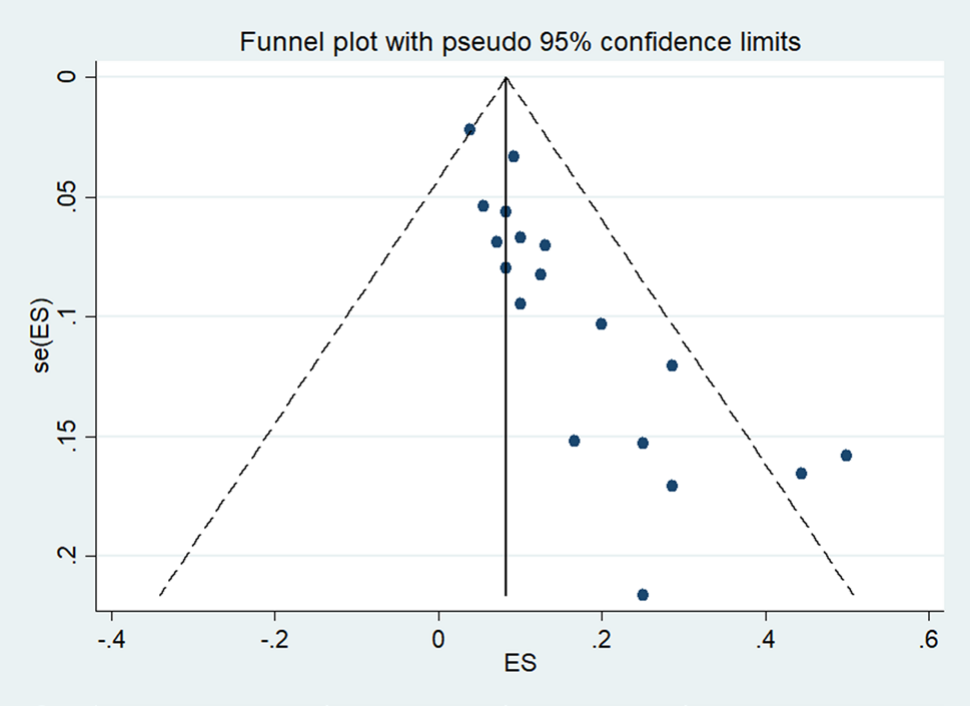
Publication bias among the included studies. Publication bias was determined using Review Manager 5.3 (The Cochrane Collaboration, London, UK) available at https://training.cochrane.org/.

**Figure 7:**
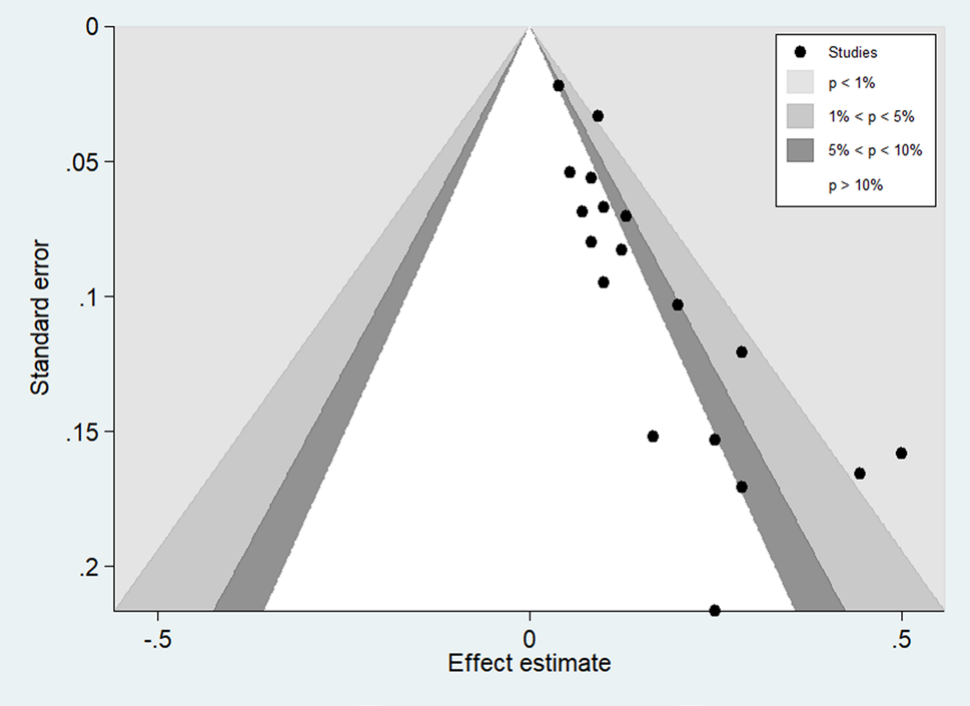
The contour enhanced funnel plot. The contour enhanced funnel plot was estimated using STATA Statistical Software version 15.0 (StataCorp. 2017. Stata Statistical Software: Release 15. College Station, TX: StataCorp LLC).

Visual inspection of the funnel plots demonstrated some small study effects that caused an asymmetric distribution of studies in the plots between the OR and SE (logOR) (Fig. 6). The asymmetric distribution of the funnel plots was quantified with Egger's test. Egger's test showed a significant asymmetric distribution due to the small-study effects across the 22 included studies (p: 0.001, coefficient: 12.5, standard error: 3.17, t: 3.95). The contour-enhanced funnel plot was further evaluated if the asymmetric distribution was due to publication bias or other factors. The results showed that most of the included studies were located in the significant area of the plot (p < 0.01), indicating that publication bias was the cause of the asymmetric distribution of the funnel plot (Fig. 7).

## Discussion

The microscopic method for identifying *Plasmodium* species is still considered the gold standard method for malaria diagnosis in clinical laboratories. However, its limitation is its low sensitivity to detect malaria parasites that are present with a low parasite density^39–41^. The sensitivity of microscopy under optimal conditions is limited to approximately 10–50 parasites/μl of blood^42^. In contrast to microscopy, PCR has the advantage of higher sensitivity and is capable of detecting less than 10 parasites/µl of blood^43–45^. In addition to low sensitivity, microscopy also has a low specificity or inability to distinguish the morphologically similar *P. vivax* and *P. ovale* malaria even by a well-trained or expert microscopist examining blood films.

This is the first systematic review and meta-analysis to quantify the misidentification of *P. ovale* as *P. vivax* by microscopic methods. The prevalence of misidentification of *P. ovale* as *P. vivax* malaria was high (11%), particularly in *P. ovale* endemic countries (13%). A previous study suggested that misidentification of *P. vivax* with *P. ovale* was likely due to the infection of *P. ovale* resulting in a low parasite density compared to that of *P. falciparum*^18^, and the misidentification of *P. ovale* as *P. vivax* is more frequent than the misidentification of *P. ovale* as *P. falciparum*^29^. A previous study included laboratories in hospitals and demonstrated that participants had much more difficulty identifying *P. ovale* with 100% failure rates, while difficulty identifying *P. malariae* (22.5% failure) and *P. vivax* (21.7% failure) was lower^46^. The difficulty in identifying *P. ovale* malaria was also observed by a study on laboratories in the United Kingdom^47^. In addition, a survey study of 19 provincial laboratories in China with a total of 168 staff members also demonstrated that *P. ovale* was likely to be misdiagnosed as *P. vivax* by microscopy^48^. The external quality assessment (EQA) conducted in Senegal, which was part of the national malaria control program (NMCP), demonstrated the misidentification of a *P. ovale* slide as *P. vivax* by experts^49^. Moreover, microscopists participating in post training on the proficiency of laboratory technicians in *Plasmodium* species identification could misidentify *P. vivax* as *P. ovale* malaria^50^. Although the prevalence of misidentification of *P. ovale* as *P. vivax* was high, the treatment of these two species with chloroquine and radical cure with primaquine to eliminate the liver stages were similar.

Imported malaria in non-endemic countries continues to be reported worldwide as international travel or immigration from endemic zones has increased^51–54^. Therefore, malaria diagnostic tests with high sensitivity and specificity to identify *Plasmodium* species among travellers are necessary. Due to the low sensitivity of RDTs to identify *P. ovale*^27,55,56^, molecular techniques such as PCR are recommended to identify *P. ovale* cases, although they have been shown to miss some *P. ovale* cases^57^. Moreover, molecular methods such as semi-nested PCR more accurately detected *P. ovale* mixed infections than microscopy^28^. With more advances in molecular methods for the detection and identification of malaria parasites, real-time PCR methods using fluorescent labels for detecting and quantifying DNA targets have been developed with high sensitivity and specificity^23,58,59^. Interestingly, multiplex real-time PCR failed to detect *P. falciparum* and *P. ovale* mixed infection in a previous study because of the high difference in the ratio between *P. falciparum* and *P. ovale* (> 1000:1)^32^. These results suggested that the misidentification of *P. ovale* as *P. vivax* in several studies might be caused by bias across the considerable prevalence of the main endemic *Plasmodium* species, by changes in the morphology of the parasite during specimen storage or treatment, or by very low parasitaemia levels. In addition, there is a strong perception that *P. vivax* is rare in subtropical Africa^60^. Therefore, the identification bias in retrospective studies of imported malaria, where the patients are considered to have acquired their infection in a subtropical African country, is because there is a clear bias in these cases to identify any non-falciparum or non-malariae case as *P. ovale* can occur. Thus, the number of *P. vivax* cases misidentified as *P. ovale* was quantified. The results showed that the misidentification of *P. vivax* as *P. ovale* in imported countries was very low (1%). This result indicated a lower possibility of *P. vivax* being misidentified as *P. ovale* (1%) than those of *P. ovale* being misidentified as *P. vivax* (11%) in imported cases.

The major concern of the misidentification of *P. ovale* as *P. vivax* in imported cases should be addressed. Therefore, the molecular method can make a decisive contribution to the identification of a less common *Plasmodium* species or two *Plasmodium* species with similar morphologies. Taking into account the high sensitivity and specificity of molecular methods for identifying malaria parasites, molecular methods are labour intensive and have a greater potential for contamination and long turnaround times for routine diagnosis, and are not convenient for use in remote settings. Updated courses and intensified training of microscopic examinations are necessary to improve the microscopic identification of *Plasmodium* species in rural health centres, where molecular techniques are unavailable.

The present study have limitations. First, limited numbers of *P. ovale* were identified and reported, as it is a neglected *Plasmodium* species. Therefore, the pooled prevalence of the misidentification of *P. ovale* as *P. vivax* malaria might not represent all misidentification that occurred. Second, several studies performed microscopy and PCR to confirm malaria infection, but could not be included in the present study as the necessary data cannot be extracted and the comparison between microscopy and PCR was either not clearly presented or not provided.

## Conclusion

Misidentification of *P. ovale* infections as *P. vivax* malaria by microscopic methods are frequent and lead to the underreported status of *P. ovale* cases worldwide. The molecular identification of *P. ovale* malaria in endemic areas is necessary to provide data for malaria elimination because a higher rate of *P. ovale* misidentification was found in endemic cases than in imported cases. In addition, updated courses and intensified training of microscopic examinations, particularly for *P. ovale* identification, are required to improve the microscopic identification of *Plasmodium* species in rural health centres and other resource-limited territories where PCR is unavailable.

## Supplementary Information

Supplementary Information

Supplementary Table 1.

Supplementary Figure 1.

## Data Availability

The datasets used during the current study are demonstrated in the present manuscript along with additional files.
