# Supplementary material for: Misidentification of Plasmodium ovale as Plasmodium vivax malaria by a microscopic method: a meta-analysis of confirmed P. ovale cases
Source: Sci Rep. 2020 Dec 11;10:21807. doi: 10.1038/s41598-020-78691-7 (PMC7733466; doi:10.1038/s41598-020-78691-7)
Supplement: Supplementary file 2 — Supplementary Table 1. [file 41598_2020_78691_MOESM2_ESM.docx]

**Misidentification of *Plasmodium ovale* as *Plasmodium vivax* malaria by a microscopic method: a meta-analysis of confirmed *P. ovale* cases**

Manas Kotepui^1*^, Frederick Ramirez Masangkay^2^, Kwuntida Uthaisar Kotepui^1^, Giovanni De Jesus Milanez^2^

^1^Medical Technology, School of Allied Health Sciences, Walailak University, Thasala, Nakhon Si Thammarat, Thailand

^2^Department of Medical Technology, Institute of Arts and Sciences, Far Eastern University-Manila, Manila, Philippines

Authors’ Email Addresses:

**^*^Corresponding Author**: Manas Kotepui; manas.ko@wu.ac.th, +66954392469

Frederick Ramirez Masangkay; frederick_masangkay2002@yahoo.com

Kwuntida Uthaisar Kotepui; kwuntida.ut@wu.ac.th

Giovanni De Jesus Milanez; gmilanez@feu.edu.ph

**S1 Table. Search term**

| **Databases** | **Search terms** | **Search date** |
| --- | --- | --- |
| MEDLINE | (Plasmodium OR malaria) AND (ovale OR P. ovale) AND (microscopy OR microscopic OR blood film OR "blood film" OR "thick film" OR "thin film") AND (PCR OR "polymerase chain reaction") | 13 August 2020 |
| Scopus | (Plasmodium OR malaria) AND (ovale OR P. ovale) AND (microscopy OR microscopic OR blood film OR "blood film" OR "thick film" OR "thin film") AND (PCR OR "polymerase chain reaction")  Search option: All fields | 13 August 2020 |
| ISI Web of Science | (Plasmodium OR malaria) AND (ovale OR P. ovale) AND (microscopy OR microscopic OR blood film OR "blood film" OR "thick film" OR "thin film") AND (PCR OR "polymerase chain reaction")  Search option: All fields | 13 August 2020 |
